# Supplementary figures and images for: Biosafety devices to control the spread of potentially contaminated dispersion particles. New associated strategies for health environments
Source: PLoS One. 2021 Aug 26;16(8):e0255533. doi: 10.1371/journal.pone.0255533 (PMC8389494; doi:10.1371/journal.pone.0255533)

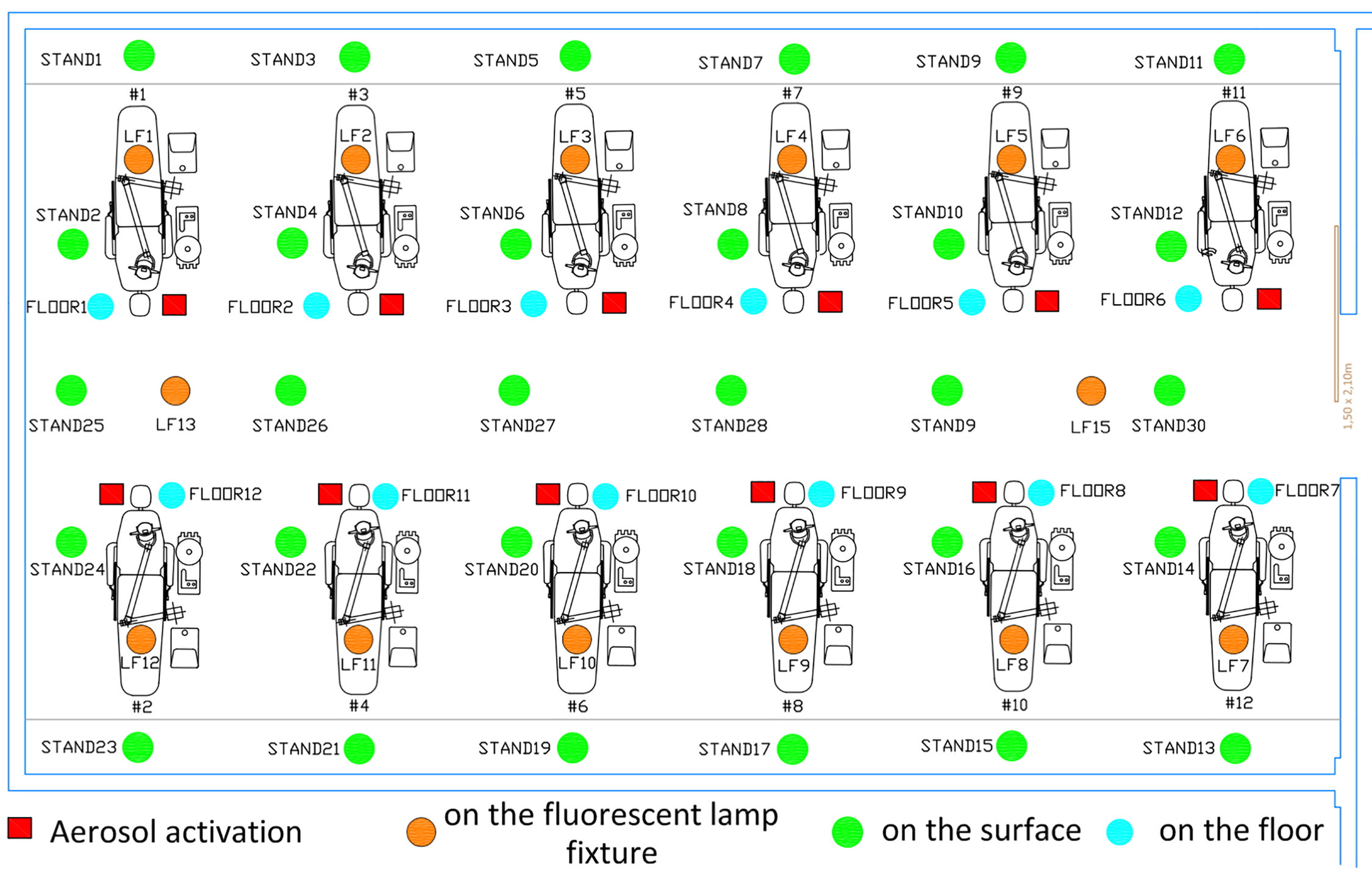

Supplement: S1 Fig — On the surface (Stand), green circles; On the fluorescent lamp fixture (LF), orange circles; On the floor (Floor), blue circles; Position of operators for activation of dental drill (aerosol activation), red squares. (TIF) [file pone.0255533.s001.tif]

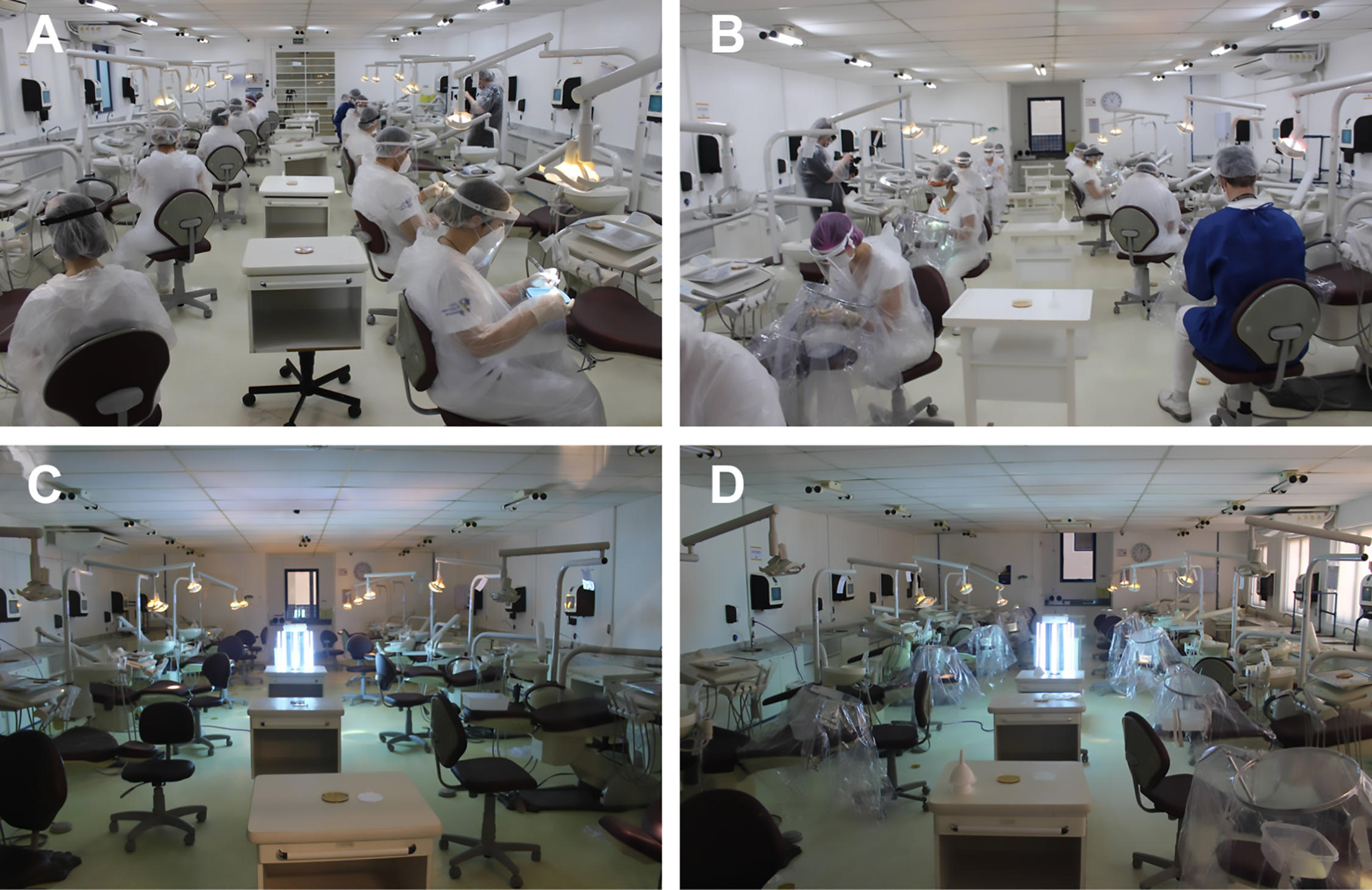

Supplement: S2 Fig — After activation, the Petri dishes were opened and remained open for 15 minutes in the pre stablished position. Clinic Positive Control Group (no barriers) (A); Clinic Individual Biosafety Barrier in Dentistry (IBBD) Group (B); Clinic UV-C Group (C) and; Clinic IBBD + UV-C Group (D). (TIF) [file pone.0255533.s002.tif]

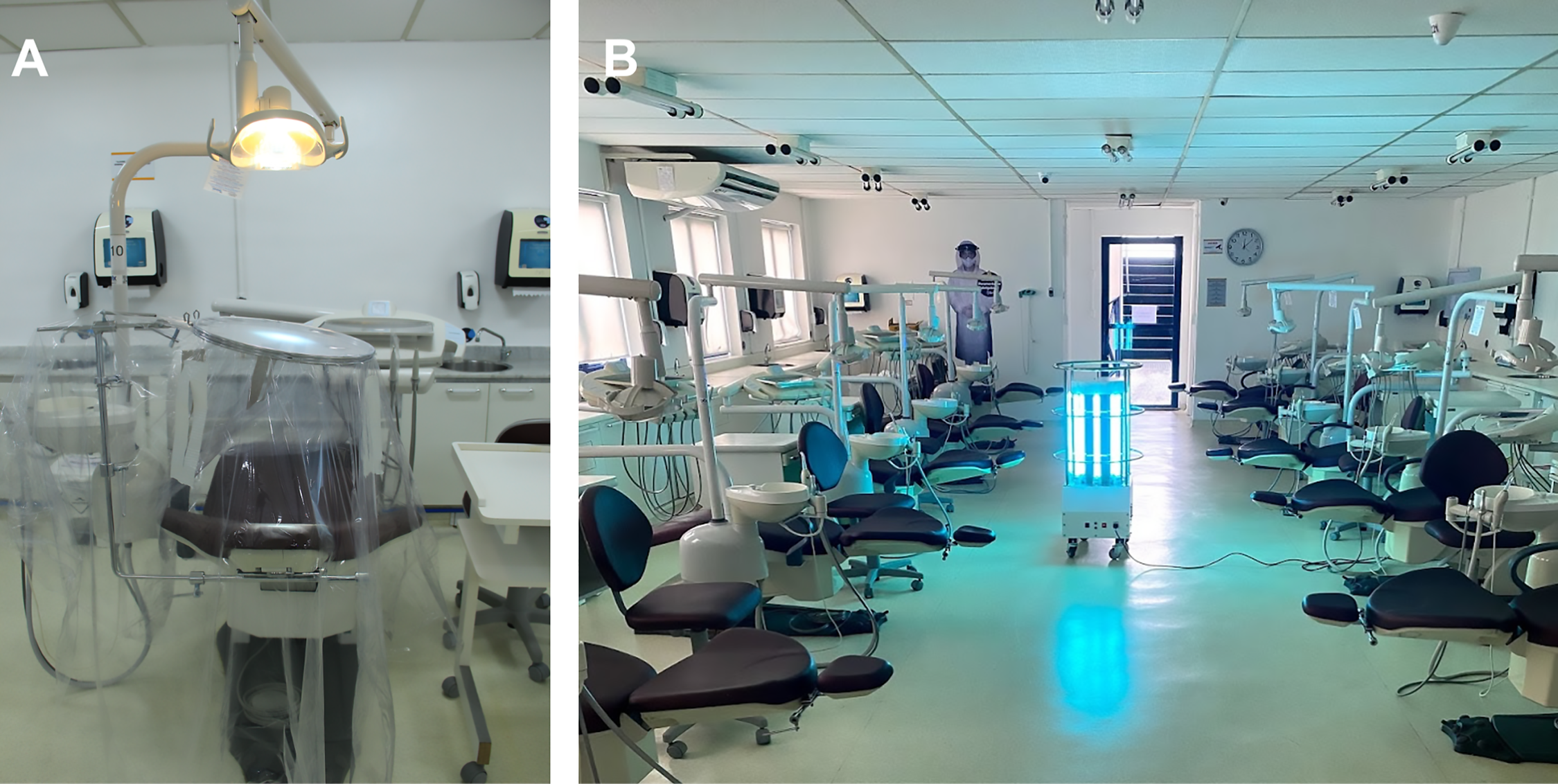

Supplement: S3 Fig — Protection barrier against droplets and aerosol is made using a metal support, with a 30 cm ring and covered by a disposable 30 microns thickness PVC film measuring approximately 1.5 x 1.5 m (A); Ultraviolet-C Device Mobile Disinfection Unit (UMDUV 2.0). The equipment is composed of 8 UVGI lamps of 95W of low-pressure mercury with 304μW/cm2 irradiance, without ozone generation (B). (TIF) [file pone.0255533.s003.tif]

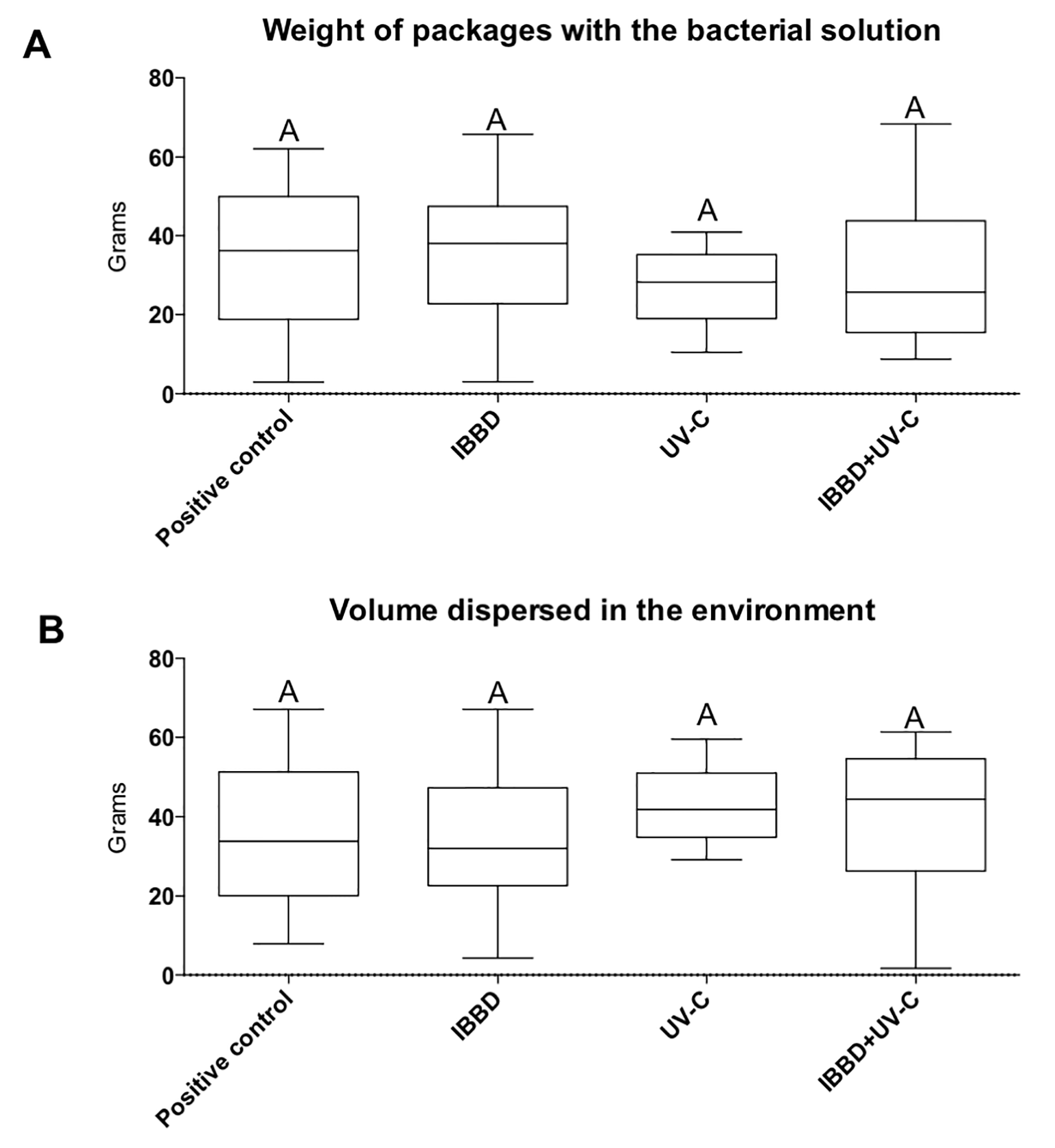

Supplement: S4 Fig — Mass (in grams) of the bacterial solution after activation of dental drill refrigeration (A) in the packages and dispersed in the environment (B) (p >0.05). (TIF) [file pone.0255533.s004.tif]

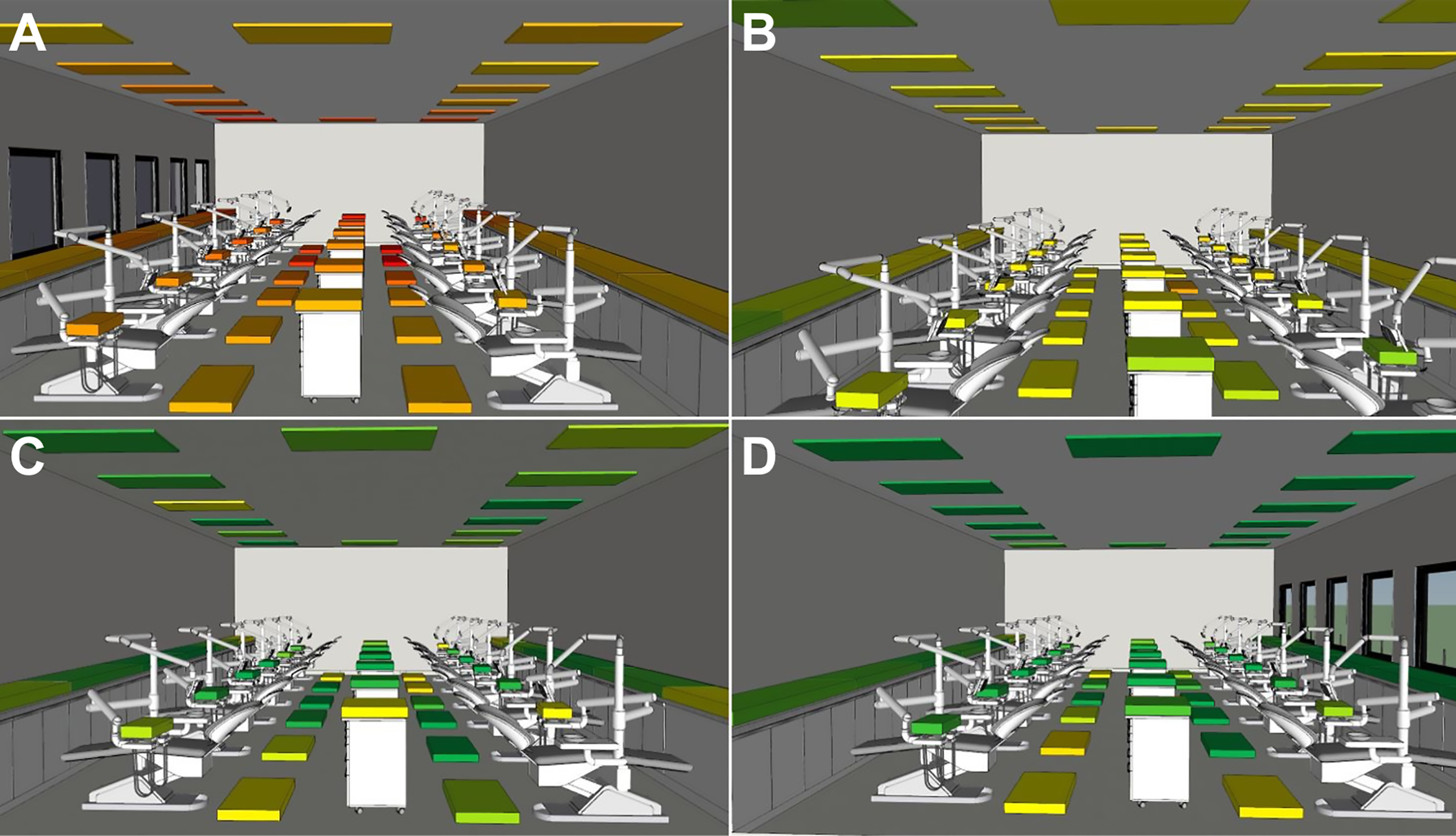

Supplement: S5 Fig — Scheme 3 D with Heat map of the results obtained from CFU counts in clinics: positive control (A); IBBD; UV-C; and IBBD + UV-C. The mean difference between the positive control group and the IBBD group was, on average, 75% (B). When using UV-C device, average CFU counts had, on average, a 93% (C) and 96% reduction in IBBD+UV-C (D). (TIF) [file pone.0255533.s005.tif]
