# Supplementary material for: Biosafety devices to control the spread of potentially contaminated dispersion particles. New associated strategies for health environments
Source: PLoS One. 2021 Aug 26;16(8):e0255533. doi: 10.1371/journal.pone.0255533 (PMC8389494; doi:10.1371/journal.pone.0255533)
Supplement: S1 Table — (DOCX) [file pone.0255533.s006.docx]

|  |  | **Positive control** | | **IBDB** | | **UV-C** | | **IBDB + UV-C** | | | **Reduction comparing with positive control (%)** | | | |
| --- | --- | --- | --- | --- | --- | --- | --- | --- | --- | --- | --- | --- | --- | --- |
| **Chair number** | **Plate area** | **CFU** | **Heat map** | **CFU** | **Heat map** | **CFU** | **Heat map** | **CFU** | **Heat map** | **IBDB** | | **UV-C** | **IBDB + UV-C** |  |
| 12 | **STAND 13** | 5450 |  | 1166 |  | 652 |  | 128 |  | 79 | | 88 | 98 |  |
| 12 | **STAND 14** | 6680 |  | 1569 |  | 624 |  | 202 |  | 77 | | 91 | 97 |  |
| 12 | **LF 7** | 5599 |  | 1591 |  | 361 |  | 47 |  | 72 | | 94 | 99 |  |
| 12 | **FLOOR 7** | 7104 |  | 1072 |  | 1357 |  | 352 |  | 85 | | 81 | 95 |  |
| 11 | **STAND 11** | 5641 |  | 1548 |  | 624 |  | 452 |  | 73 | | 89 | 92 |  |
| 11 | **STAND 12** | 6532 |  | 1251 |  | 0 |  | 198 |  | 81 | | 100 | 97 |  |
| 11 | **STAND 30** | 6977 |  | 1244 |  | 285 |  | 266 |  | 82 | | 96 | 96 |  |
| 11 | **LF 6** | 5280 |  | 1230 |  | 174 |  | 160 |  | 77 | | 97 | 97 |  |
| 11 | **LF 15** | 5440 |  | 1410 |  | 268 |  | 104 |  | 74 | | 95 | 98 |  |
| 11 | **FLOOR 6** | 5874 |  | 948 |  | 806 |  | 712 |  | 84 | | 86 | 88 |  |
| 10 | **STAND 15** | 4347 |  | 1272 |  | 267 |  | 137 |  | 71 | | 94 | 97 |  |
| 10 | **STAND 16** | 3732 |  | 1166 |  | 123 |  | 39 |  | 69 | | 97 | 99 |  |
| 10 | **LF 8** | 4241 |  | 1739 |  | 272 |  | 0 |  | 59 | | 94 | 100 |  |
| 10 | **FLOOR 8** | 7613 |  | 2884 |  | 1271 |  | 142 |  | 62 | | 83 | 98 |  |
| 9 | **STAND 9** | 4220 |  | 1230 |  | 219 |  | 1 |  | 71 | | 95 | 100 |  |
| 9 | **STAND 10** | 5577 |  | 1527 |  | 263 |  | 65 |  | 73 | | 95 | 99 |  |
| 9 | **STAND 29** | 5026 |  | 852 |  | 63 |  | 44 |  | 83 | | 99 | 99 |  |
| 9 | **LF 5** | 3987 |  | 920 |  | 276 |  | 61 |  | 77 | | 93 | 98 |  |
| 9 | **FLOOR 5** | 6256 |  | 1000 |  | 10 |  | 136 |  | 84 | | 100 | 98 |  |
| 8 | **STAND 17** | 2905 |  | 997 |  | 61 |  | 25 |  | 66 | | 98 | 99 |  |
| 8 | **STAND 18** | 3817 |  | 1421 |  | 2 |  | 0 |  | 63 | | 100 | 100 |  |
| 8 | **LF 9** | 2693 |  | 1145 |  | 1 |  | 0 |  | 57 | | 100 | 100 |  |
| 8 | **FLOOR 9** | 4708 |  | 1108 |  | 149 |  | 10 |  | 76 | | 97 | 100 |  |
| 7 | **STAND 7** | 3414 |  | 912 |  | 5 |  | 186 |  | 73 | | 100 | 95 |  |
| 7 | **STAND 8** | 5026 |  | 992 |  | 4 |  | 17 |  | 80 | | 100 | 100 |  |
| 7 | **STAND 28** | 3923 |  | 968 |  | 0 |  | 0 |  | 75 | | 100 | 100 |  |
| 7 | **LF 4** | 4453 |  | 956 |  | 8 |  | 2 |  | 79 | | 100 | 100 |  |
| 7 | **FLOOR 4** | 4178 |  | 956 |  | 5 |  | 7 |  | 77 | | 100 | 100 |  |
| 6 | **STAND 19** | 2651 |  | 1103 |  | 76 |  | 0 |  | 58 | | 97 | 100 |  |
| 6 | **STAND 20** | 2545 |  | 1272 |  | 2 |  | 0 |  | 50 | | 100 | 100 |  |
| 6 | **LF 10** | 2651 |  | 572 |  | 9 |  | 2 |  | 78 | | 100 | 100 |  |
| 6 | **FLOOR 10** | 3202 |  | 676 |  | 1 |  | 1 |  | 79 | | 100 | 100 |  |
| 5 | **STAND 5** | 4050 |  | 800 |  | 39 |  | 98 |  | 80 | | 99 | 98 |  |
| 5 | **STAND 6** | 4284 |  | 1004 |  | 2 |  | 0 |  | 77 | | 100 | 100 |  |
| 5 | **STAND 27** | 3478 |  | 904 |  | 0 |  | 0 |  | 74 | | 100 | 100 |  |
| 5 | **LF 3** | 3711 |  | 668 |  | 6 |  | 7 |  | 82 | | 100 | 100 |  |
| 5 | **FLOOR 3** | 3393 |  | 608 |  | 66 |  | 452 |  | 82 | | 98 | 87 |  |
| 4 | **STAND 21** | 2333 |  | 604 |  | 178 |  | 14 |  | 74 | | 92 | 99 |  |
| 4 | **STAND 22** | 3181 |  | 544 |  | 87 |  | 78 |  | 83 | | 97 | 98 |  |
| 4 | **LF 11** | 1739 |  | 1315 |  | 274 |  | 5 |  | 24 | | 84 | 100 |  |
| 4 | **FLOOR 11** | 2587 |  | 456 |  | 27 |  | 19 |  | 82 | | 99 | 99 |  |
| 3 | **STAND 3** | 2418 |  | 456 |  | 139 |  | 204 |  | 81 | | 94 | 92 |  |
| 3 | **STAND 4** | 3542 |  | 560 |  | 83 |  | 64 |  | 84 | | 98 | 98 |  |
| 3 | **STAND 26** | 3520 |  | 672 |  | 94 |  | 31 |  | 81 | | 97 | 99 |  |
| 3 | **LF 2** | 2948 |  | 596 |  | 127 |  | 24 |  | 80 | | 96 | 99 |  |
| 3 | **FLOOR 2** | 2820 |  | 640 |  | 517 |  | 1406 |  | 77 | | 82 | 50 |  |
| 2 | **STAND 23** | 2142 |  | 412 |  | 624 |  | 22 |  | 81 | | 71 | 99 |  |
| 2 | **STAND 24** | 2333 |  | 348 |  | 596 |  | 376 |  | 85 | | 74 | 84 |  |
| 2 | **LF 12** | 2375 |  | 560 |  | 420 |  | 7 |  | 76 | | 82 | 100 |  |
| 2 | **FLOOR 12** | 2566 |  | 376 |  | 388 |  | 876 |  | 85 | | 85 | 66 |  |
| 1 | **STAND 1** | 2078 |  | 311 |  | 388 |  | 230 |  | 85 | | 81 | 89 |  |
| 1 | **STAND 2** | 3181 |  | 536 |  | 444 |  | 149 |  | 83 | | 86 | 95 |  |
| 1 | **STAND 25** | 2375 |  | 408 |  | 612 |  | 192 |  | 83 | | 74 | 92 |  |
| 1 | **LF 1** | 1527 |  | 304 |  | 107 |  | 33 |  | 80 | | 93 | 98 |  |
| 1 | **LF 13** | 1951 |  | 668 |  | 264 |  | 11 |  | 66 | | 86 | 99 |  |
| 1 | **FLOOR 1** | 2396 |  | 176 |  | 840 |  | 720 |  | 93 | | 65 | 70 |  |
|  | **Min** | 1527 |  | 176 |  | 0 |  | 0 |  | 24 | | 65 | 50 |  |
|  | **Max** | 7613 |  | 2884 |  | 1357 |  | 1406 |  | 93 | | 100 | 100 |  |
|  | **Mean** | 3905 |  | 940 |  | 260 |  | 152 |  | 75 | | 93 | 96 |  |
|  | **SD** | 1521 |  | 466 |  | 309 |  | 257 |  | 11 | | 9 | 9 |  |
|  | ***p*** | A | | B | | C | | C | | - | | - | - |  |
